# MALBoost: a web-based application for gene regulatory network analysis in *Plasmodium falciparum*

**DOI:** 10.1186/s12936-021-03848-2

**Published:** 2021-07-14

**Authors:** Roelof van Wyk, Riëtte van Biljon, Lyn-Marie Birkholtz

**Affiliations:** 1 Department of Biochemistry, Genetics and Microbiology and the Institute for Sustainable Malaria Control, University of Pretoria, Private Bag X20, Hatfield, Pretoria, 0028 South Africa; 2 Department of Biochemistry and Molecular Biology and the Huck Centre for Malaria Research, Pennsylvania State University, University Park, PA 16802 USA

**Keywords:** Gene regulatory network, Malaria, *Plasmodium falciparum*, Machine learning, Artificial intelligence

## Abstract

**Background:**

Gene Regulatory Networks (GRN) produce powerful insights into transcriptional regulation in cells. The power of GRNs has been underutilized in malaria research. The Arboreto library was incorporated into a user-friendly web-based application for malaria researchers (http://malboost.bi.up.ac.za). This application will assist researchers with gaining an in depth understanding of transcriptomic datasets.

**Methods:**

The web application for MALBoost was built in Python-Flask with Redis and Celery workers for queue submission handling, which execute the Arboreto suite algorithms. A submission of 5–50 regulators and total expression set of 5200 genes is permitted. The program runs in a point-and-click web user interface built using Bootstrap4 templates. Post-analysis submission, users are redirected to a status page with run time estimates and ultimately a download button upon completion. Result updates or failure updates will be emailed to the users.

**Results:**

A web-based application with an easy-to-use interface is presented with a use case validation of AP2-G and AP2-I. The validation set incorporates cross-referencing with ChIP-seq and transcriptome datasets. For AP2-G, 5 ChIP-seq targets were significantly enriched with seven more targets presenting with strong evidence of validated targets.

**Conclusion:**

The MALBoost application provides the first tool for easy interfacing and efficiently allows gene regulatory network construction for *Plasmodium*. Additionally, access is provided to a pre-compiled network for use as reference framework. Validation for sexually committed ring-stage parasite targets of AP2-G, suggests the algorithm was effective in resolving “traditionally” low-level signatures even in bulk RNA datasets.

**Supplementary Information:**

The online version contains supplementary material available at 10.1186/s12936-021-03848-2.

## Background

A Gene Regulatory Network (GRN) depicts transcriptional regulation and provides an understanding of the dynamics and interaction of and between genes [1, 2]. In its simplest form, a GRN connects a “regulatory” gene (also referred to as candidate gene or effector) to its target gene (affected gene), capturing the effector *vs*. affected relationship. In principle, changes in expression of the regulatory gene will by implication then result in differential expression of the target gene sets. The construction of such a network may have its roots in either direct experimentation or modelled inferential approaches [3], allowing statistically validated inferences from gene expression data.

A GRN synthesized for a disease phenotype consists of a collection of regulators that make up a powerful dataset to understand molecular effectors allowing disease severity, progression, pathological markers and therapeutic efficacy. Such a causal molecular map is, therefore, extraordinarily useful to define observed phenotypes. Moreover, comparative network analysis is possible, where GRNs from different biological or physiological states can be statistically compared and differential GRN analysis can identify regulators with changes under disparate conditions. The use of GRNs in malaria research is sparse. However, with more and more transcriptome data being produced, particularly for the most lethal of human malaria parasite, *Plasmodium falciparum*, there is a critical need for deeper interpretation of the data and tools such as GRN analysis become imperative. Previous small-scale GRN investigations in *P. falciparum* have focused only on select genes involved in red blood cell invasion [2, 4] or the intra-erythrocytic development cycle (IDC) of the parasite [5]. Weighted co-expression networks constructed from large DNA-microarray datasets have provided insights regarding the involvements of regulators potentially affecting parasite transmission including the Apicomplexan Apetala 2 transcription factor AP2-G, histone deacetylase 1 (HDAC1) and a putative histone deacetylase (HDA1) [6].

Inferential-based GRN work has been done using machine learning based approaches for the malaria research field. Such Bayesian Network approaches have been popular tools for GRN construction in the past and remain some of the most reliable network methods to date [7]. Dynamic Bayesian Network (DBN) approaches have proven to be easily implementable frameworks for various aspects of *P. falciparum* regulatory networks [8, 9]. However, there are some key limitations of the DBNs constructed for *P. falciparum* datasets thus far: (1) only a small number of candidate and target genes have been investigated due to the complexity associated with DBN construction; and (2) networks were constructed from very small sample sets. The latter is often a challenge inherent in working with *P. falciparum* transcriptome data. In fact, the choice of algorithm for any viable GRN construction for this parasite needs to be guided by the suitability of the algorithm to handle small sample sets and allow for the evaluation of larger genes datasets.

Decision tree algorithms such as random forest (RF) trees (ensemble learning method which considers the aggregate of all trees) and gradient boosting machine (GBMs: like RFs construct multiple trees, but learn from shallow trees rather than consolidating complete trees, thus shortening the learning process) allow for expedited assessment of a large number of genes as compared to DBNs. A prominent GBM algorithm, GRNBoost2 (http://aertslab.org, [10]) was designed to increase speed and accuracy of inferred GRNs.

GRNBoost2 has previously been used to analyse, for instance, human single cell RNA-seq (scRNA-seq) with thousands of samples [10] but suffers from being computationally intensive. Since the *P. falciparum* genome is comparatively small (~ 23 Mb, with ~ 5700 genes [11]), we postulated that sophisticated tools like GRNBoost could be successfully applied to this organism, without being resource intensive. Here, a web-based application, MALBoost was developed to fulfil this function. This application executes the full Arboreto suite of tools for GRN construction. This suite consists of GRNBoost2 and GENIE3 algorithms use to construct GRNs, The application uses a user-friendly web interface to execute the algorithms from Arboreto and is suitable for *P. falciparum* gene expression datasets due to the small genome and transcriptome size.

The application intends to serve as an in silico testing platform for malaria research to investigate the regulatory roles of genes in the parasite. Construction of unique user-specific GRNs can be done with the choice of two algorithms, GENIE3 and GRNBoost2 of which the latter is recommended [12, 13]. A pre-compiled GRN constructed from 124 selected candidate genes and 5163 target genes for *P. falciparum* is also available for download from the application as a reference framework of molecular regulators in the parasite. As proof-of-concept, we use GRNboost2 results obtained from MALBoost to investigate the regulatory activity of two transcription factors with well-characterized DNA-binding sites in the *Plasmodium* genome. The data validates previously experimentally confirmed target genes of the AP2-G transcription factor [14], but also shows the depth of analyses possible with GRNs, as it expands on the number of target genes of this important regulator particularly involved in sexual commitment in the parasite lifecycle [14, 15]. The data show GRNs and tools like MALBoost could be powerful resources for the malaria research community and related parasites with similarly sized genomes by rapidly and inexpensively evaluating and inferring molecular regulators of malaria parasite biology.

## Methods

### Application architecture and core concepts

In order to make GRNBoost2 more accessible to a wider audience of researchers in the malaria field, the tools from GRNBoost2 were packaged in a user-oriented web-based application, MALBoost, with intended use in the malaria research field. This provides access to GRNBoost2, GENIE3 as well as pre-compiled networks produced from this study which are downloadable [12]. Arboreto requires Scikit-Learn and Dask for model construction [16, 17]. Scikit-Learn is a library for machine learning in Python from which the GBMs are built, while Dask is library for parallel computing that incorporates Scikit-Learn models. The application is constructed through Flask which is a microframework for web applications built in Python [18]. Flask handles request via the web interface and distributes the request to various technologies. Flask requires a Web Server Gateway Interface (WSGI) in order to handle these requests more efficiently, here Green Unicorn (Gunicorn) provides this service [19]. Since the GRN models will take time to complete the application facilitates these requests in the background. Background processing requires two components: (1) a task queuing server, and (2) an in-memory broker [20, 21]. For this particular implement Celery is used as a task queuing server system with Redis as a data broker for passing request data onto the Celery workers [22]. This also provides multiple submissions in the task queue.

The network results are temporarily stored in an SQLite database (DB) for retrieval [23]. The results are downloaded through the dispatched link. Data stored in the DB are temporarily stored for 3 days and deleted via the Flask application scheduler. This ensures that the DB does not get congested and that personal details of users and results are not stored long term. Submission data (transcriptomes submitted) are passed onto the task queuing servers which run the GRN models through the SQL DB, however the transcriptome data itself is immediately purged from the DB upon model completion. This is to ensure the privacy of the user and to not strain the storage resources of the system.

### Web-based interaction and controls for the application

The front-end architecture of the application is web-based, which gives users access to the tools without requiring any installations or hardware resources of their own. The web front end is based on a modified version of a bootstrap4 template [24], which offers free and open source templates for the use in web applications. The implementation features a submit page as well as download page (for results from a pre-compiled GRN), the submit page requires an email address and will mail job status responses to the user. The application is deployed on the University of Pretoria Centre for Bioinformatics and Computational Biology servers (http://malboost.bi.up.ac.za). The application runs in a CentOS [25] virtual environment (VM).

### Implementation of MALBoost in understanding gene regulation of candidate transcription factors

A panel of 49 RNA-seq samples of *P. falciparum* sexual and asexual blood stage development (van Wyk, in preparation) were used to construct a GRN in MALBoost using GRNBoost2 default parameters. The ApiAP2 transcription factor AP2-G has previously been mapped to its cognate binding sites in the parasite genome using chromatin immunoprecipitation with next-generation sequencing (ChIP-seq) and the effect of knock down of AP2-G on the transcriptome was measured [14], making this factor an ideal candidate for the initial investigation of the capabilities of MALBoost. The output file generated from MALBoost, that contained the 5142 probed genes with their associated importance indicated, was subsequently used to inform a threshold for the most significant target genes (either importance > 30 or the top 100 predicted regulated genes. The inferred targets were probed against genes that were enriched for AP2-G binding within 2 kbp upstream of their coding sequences [14]. Within these experiments, the ChIP-seq peaks for a second factor (AP2-I) were also investigated as validation for specific localization of AP2-G, these peaks were then subtracted to yield to uniquely bound genome regions for AP2-G. The significance of the occurrences of either factor binding upstream of the inferred targets were determined using a two-tailed Fisher’s exact test and results were visualized using the ggplot2 package in R [26]. Transcript abundance of the top target genes were sourced from PlasmoDB [11] for data pertaining to expression of genes in sexually committed schizonts [27], invasion pathway gene knockouts [28] and expression in asexual stages, gametocytes or ookinetes [29] in Additional File 1.

## Results

### Intended use

**Fig. 1:**
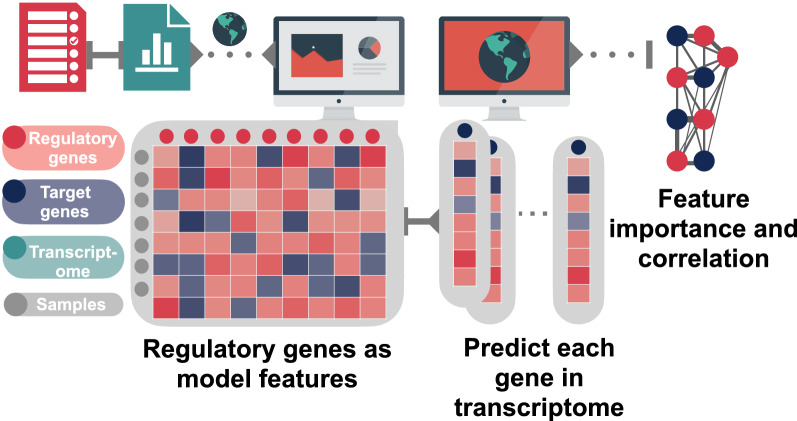
MALBoost user overview. Users can submit their transcriptomes along with a list of regulatory gene names to http://malboost.bi.up.ac.za and select from the two algorithms available for analysis

GRNs constructed through supervised machine learning algorithms such as GENIE3 and GRNBoost2 offer a fast and reliable tools for GRN research [12, 13]. However, these tools often require familiarity with the Python programming language and additional resources on which to run the analysis. MALBoost offers malaria researchers easy access to these machine-learning tools through a user-friendly, web-based application framework. Researchers can submit their own transcriptomic data for de novo network construction or download a pre-compiled and validated network built for *P. falciparum* that can be used as reference framework. Users can submit their transcriptomic data along with a list of regulatory gene names to the web-application with the selection the algorithm to be use in the analysis. The output from the algorithm along with Pearson correlations between regulatory and target genes (Fig. 1). These results are the network “flat-file” which can be interpreted with various software or programming languages. Cytoscape is recommended for easy interpretation of networks and construction of network figures. Cytoscape also hosts other secondary network analysis [30].

### MALBoost architecture and interface

**Fig. 2:**
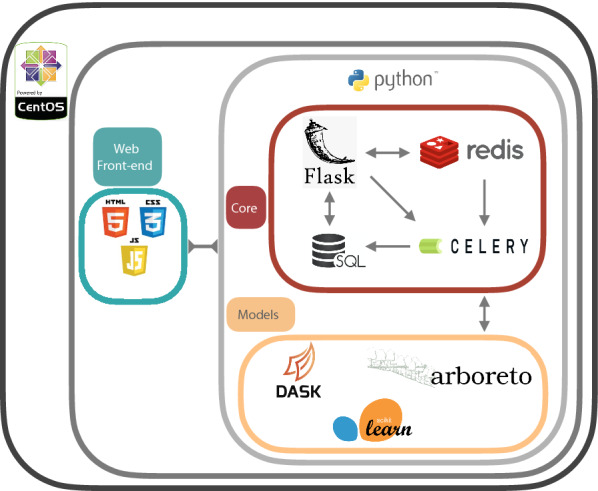
Internal architecture of MALBoost web-based application. The application runs on a CentOS virtual machine (VM). Python formulates the core coding language of the application, running everything from Flask to task queue servers and GRN model implements. The Redis data broker passes data to the Celery queuing server, which tasks individual workers with executing the model construction. A selection of either GENIE3 or GRNBoost2 is offered, for more on the models refer to [12]. Transcriptome and regulatory list data are passed to the Celery worker environment via the SQLite DB. This data is subsequently deleted upon completion of the GRN construction, the results from the GRN is stored in the DB for a period of 3 days. Once model construction is completed a download link is provided to the researcher. The web front-end is rendered via HTML, CSS and JavaScript

The application is built in a Python-based Flask microframework, giving easy access to the Arboreto suite of tools [12] which runs on a CentOS VM. The architecture of the application is shown in Fig. 2, illustrating the various technologies involved.

**Fig. 3:**
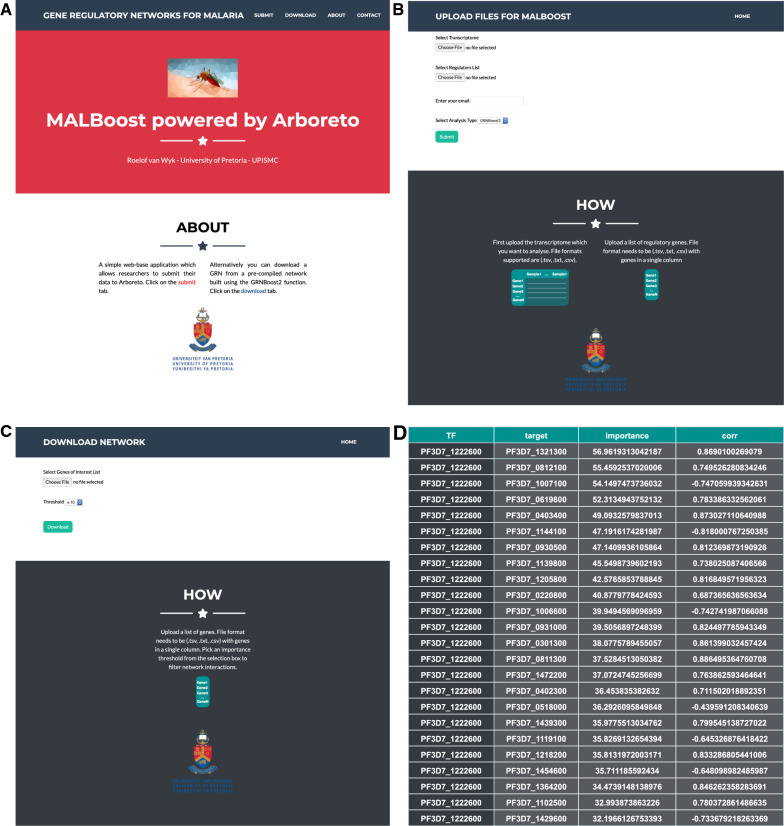
MALBoost web-based application for GRN construction in *P. falciparum*. **A** Website home page with navigation bar and description of the application. Contact detail, about and a how to guide is found on the home page along with reference material to the GRNBoost2 algorithms paper http://malboost.bi.up.ac.za. **B** The submit tab where researchers can construct their own GRNs base either on GRNBoost2 or GENIE3. Submission of transcriptome as well as a list of candidate genes (suspected in a regulatory role) is supplied as either tsv, txt or csv format files. **C** Download tab where researchers can download results based on a pre-compiled GRN. Researchers supply a list of gene IDs which they are interested in and apply an importance threshold which will download the resulting network. **D** Results format from the network (either constructed or pre-compiled). *TF* transcription factor or candidate gene, *target *target gene, *importance* importance value assigned by the selected model, *corr*  Pearson correlation of the interaction

Interfacing with the application occurs via a web portal (http://malboost.bi.up.ac.za); most modern browsers who support HTML5 should function well with the application and Google Chrome and Safari have been validated for this application. The home page of the application contains general information regarding the application as well as a navigation bar for quick navigation to functions (Fig. 3A). Contact information and reference material for the algorithms used in GRN construction may be found on the home page. Under the submit tab, the user will be re-directed to the GRN construction page with a choice of GRNBoost2 or GENIE3 (Fig. 3B). Two files are required from the user: a transcriptome file and a regulatory list file. The transcriptome file contains expression values from the user transcriptome with *Plasmodium* gene IDs as the index and samples as the column headers. Decision tree based algorithms such as GBMs are often not sensitive to normalization, however normalization is recommended and should be performed by the user prior to submission of their data [12]. The regulatory list file contains a list of candidate genes (gene IDs) the researcher has deemed relevant in a regulatory role and will be evaluated as such. This list has one gene ID per line of the file and most importantly these genes must be present in the transcriptome file as their values are required. Both files must be in the format of either csv, txt or tsv. Under the download tab, the user can download a pre-complied GRN constructed with GRNBoost2 for *P. falciparum* (Fig. 3C). A list of gene IDs of interest to the research must be supplied in previously described formats. These genes may be either candidate or target genes, the network will filter on both categories. An importance threshold is also required, which the user can assess through a drop-down tab. This will filter the network for interactions greater and equal to the set value. An example of the output data for either the submit or download tabs is shown in Fig. 3D, and includes the target genes obtained associated with importance values of the interactions and Pearson correlations for each interaction in the network. The submission web page also includes a ‘how’ section where instructions for preparing datasets for submission are provided.

### MALBoost usage in GRN construction for transcriptional regulator

**Fig. 4:**
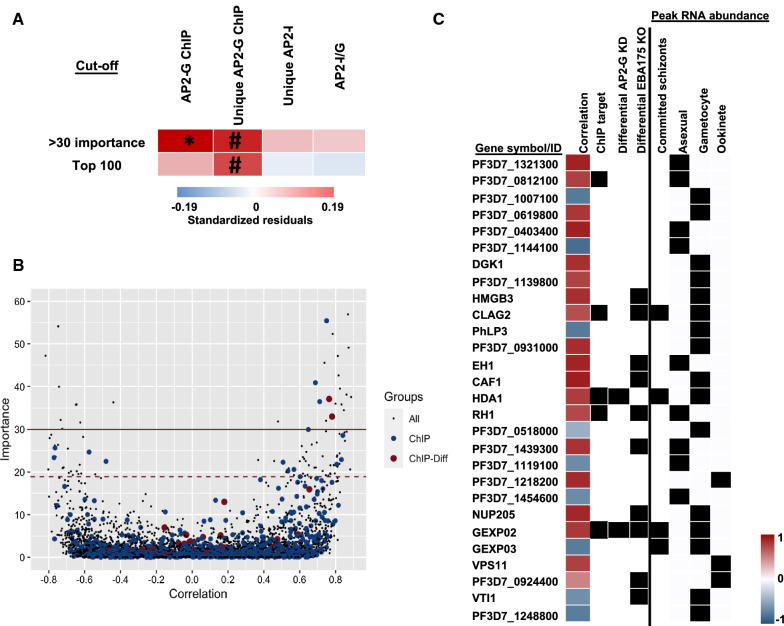
MALBoost results for AP2-G GRN. **A** The number of ChIP-targets for AP2-G and AP2-I that were within the top putative target genes of AP2-G either by importance or using top interactors were tested for significant overrepresentation using a two-tailed Fisher’s exact test (*#P* < 0.1, *P < 0.05). **B** Distribution of importance over Pearson correlation for the investigated genes (5142) with ChIP-targets (ChIP) and ChIP-targets that were differentially transcribed following AP2-G knock down (ChIP-diff) highlighted. Solid line =  > 30 importance threshold, dashed line = Top 100 interactors. **C** Genes above the 30 importance threshold were ordered by decreasing importance and Pearson correlation shown along with hits in AP2-G ChIP-seq, differential transcripts in the AP2-G knockdown, EBA175 KO, and the stage at which the transcripts peak in abundance (Additional File 1)

To validate the accuracy of the tool, MALBoost was interrogated with a known gene regulator, the AP2-G transcription factor, which was used as candidate and for which the targets were then probed in the tool. AP2-G is a known regulator of sexual commitment in *Plasmodium* parasites [15, 31] and was found to bind upstream of a specific subset of *P. falciparum* genes and associated with an increase in the transcript abundance of these genes [14]. Here, a GRN was constructed from 49 RNA-seq samples encompassing both asexual and sexual blood-stage development (van Wyk, in preparation) and the target genes of AP2-G were extracted from the GRN output (Fig. 4). Using > 30 importance as a highly significant guided threshold resulted in the identification of 28 putative targets, of which five have empirical ChIP-seq data showing their promoter regions are bound by AP2-G. This constitutes a significant overrepresentation (*P* < 0.05) of known AP2-G targets bound in sexually committed ring-stage parasites and two of these genes were also differentially expressed following AP2-G genetic perturbation (Figs. 4A and 3B). Comparatively, using Pearson correlations to investigate showed the 28 most correlated transcripts with AP2-G over the full transcriptome only contained three ChIP targets and none were perturbed following AP2-G knock down. The top 100 interactions included a further 11 AP2-G ChIP targets, but these were not significantly enriched above a random chance of occurrence (Fig. 4B). Interestingly, targets that were bound uniquely by AP2-G rather than in overlapping regions with a second transcription factor, AP2-I, were also overrepresented although not quite statistically significant (*P* < 0.1). However, targets of AP2-I either shared with AP2-G or unique to AP2-I were not overrepresented in the putative targets (Fig. 4A). These results suggest that MALBoost could pick out some directly regulated transcripts within bound target genes of transcription factors.

While the AP2-G bound genes are of interest for direct genetic regulation by the AP2-G transcription factor, probing into the top target genes (> 30 importance) of AP2-G also yield interesting implication for downstream regulation. In addition to the five directly-bound AP2-G ChIP targets, a further seven of the 28 putative targets were transcriptionally affected following a genetic knock-out of EBA175 (PF3D7_0731500*)* (Fig. 4C), an AP2-G ChIP target which is essential for parasite invasion [28]. Furthermore, the putative targets were primarily (16/28) transcriptionally abundant in gametocytes (Fig. 4C) the stage in which AP2-G is expected to regulate gene expression and it is possible that a factor downstream of AP2-G also regulates these targets directly, i.e., an epigenetic regulator (HDA1, PF3D7_1472200) or post-transcriptional regulator (CAF1, PF3D7_0811300). This suggests that MALBoost was able to find many real targets of sexually committed parasites even in bulk RNA datasets.

## Discussion

The MALBoost web application offers a user-friendly interface for malaria researchers to perform in silico experimentation based on established transcriptional data as well as newly generated data. Utilization of a Python-based Flask microframework ensures easy deployment and the combination of Redis and Celery technologies provide a powerful backend capable of running the computationally intensive Arboreto suite. The core software of the application (Arboreto and the recommended use of GRNBoost2) has shown tremendous promise within the field of cancer research [10, 12] and should translate well to use with the comparatively small genome and collective datasets in the malaria research field. Here a web-based application of this technology was built and applied to extant malaria transcriptomic and ChIP-seq datasets.

The ApiAP2 sequence-specific family of DNA binding proteins provide some of the only probable evidence of transcriptional regulation in *P. falciparum* parasites. The AP2-G transcription factor is one of the most critical proteins for study as it is essential for progression into sexual differentiation. Previous data also show that this protein directly binds the nuclear genome at very specific sites [14, 15] and influences the transcript abundance of specific genes. Within the very narrow subset of genes that are both directly bound and putatively regulated by this transcription factor, the application showed 5/28 genes could be predicted by transcriptional profile in bulk RNA-seq datasets alone. Interestingly, while this represents an overrepresentation of AP2-G targets in the predicted dataset, the algorithm did not identify over-representation of AP2-I in the predicted targets of AP2-G, despite 35% of AP2-G genome regions also being bound by AP2-I [14]. In addition, probing into the unbound predicted target genes also provided interesting results as seven more were possibly indirectly affected, as they were transcriptionally perturbed following genetic knock out of EBA175 (PF3D7_0731500), a gene regulated by AP2-G [28]. Overall, these results suggest MALBoost can discern direct and indirect effects of transcriptional regulators within a complex sample of transcriptome datasets and provides independent analysis of transcriptional variation that can be explained by the expression of a singular transcriptional regulator within complexly regulated gene sets.

## Conclusions

The use of GRN analysis in *Plasmodium* research has not been frequent while most fields have benefitted from this line of inferential reasoning. Many experimental investigations regarding regulatory mechanism and co-expression studies have been conducted, but the utilization of GRNs remain scarce. Here, GRN research has been made more accessible to the malaria field through MALBoost. Not only does the application allow for de novo construction of GRNs, but access to a pre-compiled network. The main intended use of this applications would be to assist in researcher “think tanks” or “brainstorming sessions” whereby researchers prioritize genes they which to experimentally investigate through construction of custom GRNs. This effectively creates a simulation environment for quick testing and experimental prioritization strategies. In the case of AP2-G, the data highlighted genes bound in ChIP-seq that can be investigated for the functional importance of AP2-G binding in transcriptional activation as well as possible downstream regulators of gametocytogenesis.

MALBoost offers an easy-to-use interface for researchers to perform intricate GRN analysis. The framework allows for two types of GRN construction, GRNBoost2 and GENIE3 from the Arboreto library as well as a pre-compiled network for download that can be used as reference framework. In an example of use of the tool, target validation of AP2-G shows significant enrichment in the network constructed, validating the accuracy and depth of analysis possible with MALBoost.

## Supplementary Information

**Additional file 1.** AP2-G GRN network composition and validation.

## Data Availability

http://malboost.bi.up.ac.za, https://github.com/roelof89/malboost.

## Abbreviations

ChIP-seq: Chromatin immunoprecipitation sequencing
CSS: Cascading style sheets
CSV: Comma separated values
DB: Database
DBN: Dynamic Bayesian Network
GBM: Gradient boosting machine
GRN: Gene regulatory network
HTML: Hypertext mark-up language
RF: Random forest (trees)
SQL: Structured query language
TXT: Tab-delimited file
TSV: Tab separated values
VM: Virtual machine
WSGI: Web Server Gateway Interface
